# Hematological parameters of type 2 diabetic adult patients at Debre Berhan Referral Hospital, Northeast Ethiopia: A comparative cross-sectional study

**DOI:** 10.1371/journal.pone.0253286

**Published:** 2021-06-14

**Authors:** Mesay Arkew, Tilahun Yemane, Yordanos Mengistu, Kabtamu Gemechu, Girum Tesfaye

**Affiliations:** 1 Department of Medical Laboratory Sciences, College of Health and Medical Sciences, Haramaya University, Harar, Ethiopia; 2 Faculty of Health Sciences, School of Medical Laboratory Sciences, Institute of Health, Jimma University, Jimma, Ethiopia; Oregon State University, UNITED STATES

## Abstract

**Background:**

Diabetes is a global public health problem and associated with metabolic, cellular, and blood disturbances. Hematological changes have been reported in diabetes and play a major role in diabetes-associated complications. However, reports are contradicting and data on hematological parameters of type 2 diabetic patients in the study area are scarce. Therefore, the aim of this study was to assess the hematological parameters of type 2 diabetic adult patients at Debre Berhan Referral Hospital, Northeast Ethiopia from May 01 to June 30, 2020.

**Methods:**

A comparative cross-sectional study was conducted on 268 (134 type 2 diabetic patients and 134 controls) study participants selected by systematic random sampling technique. Socio-demographic, behavioral, and clinical data were collected using a structured questionnaire and checklist. Ethical approval was obtained from Jimma University. All phase of quality assurance was maintained. Hematological parameters and blood glucose levels were determined using UniCel DxH 800 (Beckman Coulter, USA) and Biosystems A25 (Costa Brava, Spain) analyzers, respectively. Independent t-test, Mann–Whitney U-test, correlation, and logistic regression were used during data analysis. P-value <0.05 was considered as statistically significant.

**Results:**

The current study found that total white blood cell count, absolute counts of neutrophil, lymphocyte, eosinophil, and basophil, red blood cell distribution width, platelet count, and mean platelet volume were significantly higher in type 2 diabetic patients as compared to the control group (*P*<0.05). On the other hand, the mean hemoglobin was significantly lower in type 2 diabetic patients than the control group (P = 0.007). Anemia was found in 17.9% of type 2 diabetic patients. Longer duration of diabetes (AOR = 3.05, 95% CI = 1.12–8.34) and milk consumption (AOR = 4.60, 95% CI = 1.50–14.00) were significantly associated with anemia.

**Conclusion:**

This study showed a statistically significant variation in some hematological parameters of type 2 diabetic patients compared to control group. Anemia among type 2 diabetic patients was found to be a mild public health problem. Therefore, routine screening of hematological parameters should be considered for proper management of type 2 diabetic patients. Close attention should also be given to the duration of diabetes and dietary practice.

## Introduction

Diabetes mellitus (DM) is a group of metabolic disorders characterized by chronic hyperglycemia with abnormalities in the metabolism of carbohydrate, lipid, and protein resulting from defects in insulin secretion, action, or both [1]. The global burden of diabetes among adults has dramatically risen from 108 million in 1980 to 463 million cases of diabetes and 4.2 million deaths in 2019 [2]. More than three-quarters of the global diabetes burden exists in low and middle-income countries and the number of people with diabetes by 2045 is estimated to rise to 700 million worldwide [2]. Sub-Saharan Africa is experiencing a markedly increased prevalence of diabetes. According to the 2019 report of the International Diabetic Federation, 19.4 million (4.9%) adults in the Africa Region and 1.6 million (3.2%) adults in Ethiopia had diabetes [2].

Uncontrolled DM is associated with multiple disorders including metabolic, cellular, and blood disturbances leading to vascular complications [3]. Type 2 diabetes (T2DM) is a part of the metabolic syndrome that comprises dyslipidemia, obesity, hypertension, and changes in hematological parameters [4]. Hematological changes encountered in T2DM patients include changes in the function, structure, and metabolism of red blood cells (RBCs), white blood cells (WBCs), platelet (PLT) and the coagulation systems [5]. These changes may manifest as immunological and coagulation problems, and anemia characterized by a decrease in the RBC count, hemoglobin (Hgb) and hematocrit (Hct) level as compared to non-diabetic individuals [6]. Anemia is a common hematological change in patients with T2DM and often unrecognized, and estimates of its prevalence vary widely [7–9].

Hematological changes in diabetes can be caused by several factors including increased production of reactive oxygen species (ROS) and the formation of advanced glycation end products (AGEs) as a result of the long-term hyperglycemia. Increased production of ROS resulting in oxidative stress, which is implicated in tissue damage and hematological changes such as RBC dysfunction, PLT hyperactivity, and endothelial dysfunction [10–11]. These hematological changes may lead to complications such as anemia, and a state of hypercoagulability, and contribute to cardiovascular disease (CVD) in diabetic patients [12]. Another mechanism is insulin resistance, which is associated with endothelial dysfunction, increased levels of inflammatory markers, and PLT hyperactivity, which accelerates vascular complications in T2DM patients [11].

There has been renewed interest in hematological parameters such as WBC, red blood cell distribution width (RDW), mean platelet volume (MPV), platelet distribution width, and platelet count, as predictors of endothelial dysfunction and inflammation in T2DM [13,14]. An increased WBC count is a classical marker of inflammation and evidence from epidemiological studies suggests an association between WBC count and diabetes risk [15]. Platelets play a vital role in the integrity of normal homeostasis and MPV is the marker for its function [16]. Diabetes is complicated by accelerated atherosclerosis and platelet activation plays a key role in inflammation and the atherothrombosis process contributes to the development of CVD in a patient with T2DM [17]. Mean platelet volume reflects changes either in platelet stimulation or the rate of platelet production and increased MPV has been observed in diabetes patients with coronary heart disease, nephropathy, and retinopathy [18].

Generally, hematological changes have been observed in T2DM patients. Current guidelines on the management of diabetes do not recommend periodic follow-up monitoring of hematological parameters. Although studies regarding the hematological parameters of diabetic patients done in different areas came up with a range of contradictory reports. Some studies showed that there is no statistically significant difference between diabetic patients and healthy controls with respect to RBCs indices [19–21], WBC count, and platelet count [14,19], while the other study showed that RBC, WBC, and PLT indices are significantly higher in diabetic patients than controls [22]. Others reported that RBCs indices except RDW are significantly lower, whereas WBC and PLT indices are significantly higher in the diabetic group than the control group [23,24]. Moreover, there is limited information in Ethiopia, particularly in the study area regarding hematological parameters in T2DM. Parameters obtained from hematologic analyzers can provide insight into changes that occur in hematological indices such as WBC, Hgb, Hct, RBC, PLT, RDW, MPV, and other parameters. Analysis of these parameters could contribute to the following-up of the development of degenerative complications in diabetes [25]. Therefore, this study was aimed to assess hematological parameters of type 2 diabetic adult patients in comparison with screened blood donors at Debre Berhan Referral Hospital, Northeast Ethiopia.

## Materials and methods

### Study design, period and area

A comparative cross-sectional study was conducted from May 01 to June 30, 2020 at the Chronic Care Clinic of Debre Berhan Referral Hospital. The hospital is found at Debre Berhan town, located 130 km northeast of Addis Ababa, the capital city of Ethiopia at an average elevation of 3000meters [26]. In Debre Berhan, two hospitals, three health centers, and seventeen private clinics provide healthcare services. Debre Berhan Referral Hospital was established 76 years ago and provides comprehensive healthcare services to a catchment population of more than 2.4 million [27]. The hospital has been providing teaching, research, and community service. At the time of the study, the diabetes follow-up clinic at Debre Berhan Referral Hospital gives service to more than 2,100 (900 type 1 DM and 1200 T2DM) diabetic patients on regular follow‒up.

### Study participants

All adult T2DM patients attending in chronic care clinic of Debre Berhan Referral Hospital were included in the study. In addition, age and gender-matched healthy blood donors at Debre Berhan blood bank were involved as a control group. On the other hand, patients who already had any chronic diseases like cardiac, renal and liver disease, patient with HIV/AIDS, asthmatic patients, patients who were on hormone therapy like erythropoietin, insulin, taking hematin factors, anticoagulant therapy, statins, antihypertensive treatment, smoker, alcoholics, pregnant women, those patients below the age of 18 and above 65 years were not engaged in this study.

### Sample size determination and sampling technique

The sample size was calculated based on two population mean formula using G*-Power statistical free software version 3.1, by considering the following assumptions: 95% confidence level (2-tailed, α = 0.05), 80% power (β = 0.20), the ratio of sample size (T2DM/control) was 1:1, effect size (d) was 0.36 and 10% non-respondent rate. The mean and standard deviation (SD) [mean ± SD] of absolute neutrophils (10^3^/μL) for T2DM and control groups were taken from a study conducted in Gondar [23], 3.57±1.46 for T2DM and 3.11±1.04 for the control group. The sample size was determined to be 134 for each group and a total of 268 study participants were included in this study. A systematic random sampling technique was used.

### Data collection and laboratory methods

Data related to socio-demographic, and behavioral characteristics were collected using a pretested structured questionnaire through face-to-face interviews. Clinical variables including duration of diabetes, type of anti-DM drug, and fasting blood glucose level for the last two months were abstracted from the diabetic patients’ medical records using a checklist. Participants fasting blood glucose reading for at least three months including the current reading was used for computing the average blood glucose level. Blood pressure (BP) was taken by clinical nurses using an analog sphygmomanometer and stethoscope. Measurements were taken from the upper arm with the hand at the heart level after the patient had been sitting for more than 5 minutes. The data regarding anthropometric variables such as height (to the nearest centimeter without shoes), weight (to the nearest 0.1 kg), and waist circumference (WC) (taken midway between the lowest rib and the iliac crest) were collected according to the anthropometric measurements protocol. Body mass index (BMI) was calculated as weight in kilograms divided by height in meter squared. After an interview, review of records, anthropometric, and BP measurement was completed by trained clinical nurses, the study participants were sent to a laboratory where a blood sample was collected for determination of FBG, and hematological parameters.

Six-milliliter of venous blood sample (2ml in a serum separator tube and 4ml in EDTA tube) was collected from each T2DM patient by laboratory professionals after overnight fasting. On the other hand, four milliliters of venous blood were collected into EDTA test tube from the control group (blood donor) at the time of the donation. A Serum prepared from a serum separator tube was used to determine FBG. Fasting blood glucose was estimated by following the glucose oxidase method [28], using Biosystems A25 (Costa Brava, Spain) automated chemistry analyzer according to the manufacturer’s instructions. Complete blood cell count (CBC) was analyzed using UniCel DxH 800 (Beckman Coulter, USA) automated hematology analyzer using the coulter counting, spectrophotometry, and VCSn technology [29]. Fasting blood glucose, hematological parameters (RBC, Hgb, Hct, MCV, MCH, MCHC, RDW, WBCs, absolute lymphocytes, monocyte, basophil, eosinophil, neutrophils, platelet count and MPV) were analyzed at Debre Berhan Referral Hospital laboratory and recorded for each study participants using laboratory result registration form.

### Data quality assurance and management

To ensure the quality of the data, preanalytical, analytical, and post-analytical precautions were taken. The English version of the questionnaire was translated into the local language, (Amharic) and re-translated to the English version for its accuracy and consistency. The questionnaire was pretested and training was given for the data collectors before the actual data collection started. Manufacturer instructions and standard operating procedures were strictly followed during specimen collection and all other laboratory procedures. Anthropometric and BP measurements were taken twice and the average value was used for data analysis. Control materials were used for the hematology analyzer and glucose analysis. The blood samples were processed within 2 hours of specimen collection. A blood film examination was performed for suspected flags in the analyzer. Completion, and clarity of the collected data were checked regularly and the results were properly recorded, transcribed, and reviewed.

### Statistical analysis and interpretation

Data were checked for their completeness and consistency and entered into Epidata version 3.1 (Epidata Association, Odense Denmark) and exported to Statistical Package for Social Sciences (SPSS) version 25 software (IBM Corporation, USA) for analysis. The normality of data distribution was checked using histogram, and Kolmogorov-Smirnov test. The results were reported as frequency and percentages for categorical variables, mean ± SD for normally distributed continuous variable and median with interquartile range (IQR) for continuous variables with skewed distribution. Statistical differences between the groups were determined by the chi-square test for categorical variables. The comparison of hematological parameters between diabetic and control participants were done by independent t-test for normally distributed data and Mann-Whitney U test for non-normally distributed data. The correlation of hematological parameters with independent variables was assessed by Pearson’s correlation for normally distributed data and Spearman’s correlation (rho) for non-normally distributed data. Bivariate and multivariate logistic regression analysis was conducted for categorical dependent variables. Hemoglobin was adjusted for altitude based on the World Health Organization (WHO) recommendation for the diagnosis of anemia (Hemoglobin,13 g/dl (men) or 12 g/dl (women) [30]. Crude odds ratios (COR) and adjusted odds ratios (AOR) with 95% confidence intervals (CI) were used to see associations of predictors and outcomes. In any condition, P-value <0.05 was considered as statistically significant.

### Ethical consideration

Ethical clearance was obtained from the Institutional Review Board (IRB) of Jimma University, Institute of Health with letter protocol number IRB000138/2020. A support letter from the School of Medical Laboratory Science of Jimma university was submitted to Debre Berhan Referral Hospital. Permission was also obtained from Debre Berhan Referral Hospital and Debre Berhan blood bank. Written informed consent was obtained from each participant after a clear explanation of the purpose, the procedure, benefits, possible discomfort of the study, and the right to voluntary participation was given. To ensure confidentiality of data, the study participants were identified using codes instead of individual identifiers and unauthorized persons were not able to access the collected data. Results with hematological abnormalities were communicated to the physicians who were working in the chronic care clinic for proper management of the patients. For the sake of ethical issues, the control groups (blood donors) were screened in Debre Berhan blood bank.

## Results

### Demographic, anthropometric, and clinical characteristics of study participants

A total of 268 (134 T2DM patients and 134 controls) study participants were included in this study. The T2DM patients and control groups did not differ in terms of age and sex (p>0.05). Majority of the study participants 85 (63.40%) were males for both T2DM patients and controls. The mean age (mean ±SD) was 43.08±9.30 for T2DM patients and 42.71±8.60 years for controls. Out of the total study participants, around 63 (47.00%) and 76 (56.70%) have a higher educational level for T2DM and controls, respectively. About 113 (84.30%) and 107 (79.90%) were from urban residences for T2DM and controls, respectively. Regarding the anthropometric and clinical characteristics of the study participants, statistically significantly higher values of WHR (p<0.001), WC (p<0.001), BMI (p<0.001), systolic BP (p<0.001), and diastolic BP (p = 0.002) were observed in T2DM patients compared to controls. The median of FBG levels was 159.20 (136.00–185.00) and the median duration of DM since diagnosis was 7.0 (4.0–9.0) years in T2DM patients (Table 1).

**Table 1:** Socio-demographic, anthropometric, and clinical characteristics of type 2 diabetic adult patients and control group, at Debre Berhan Referral Hospital, Northeast Ethiopia, 2020 (n = 268).

Variables		T2DM (n = 134)	Control (n = 134)	P-value
Age (years), mean ± SD		43.08±9.30	42.71±8.60	0.734
Sex, n (%)	Male	85 (63.40)	85 (63.40)	1.00
	Female	49 (36.60)	49 (36.60)	
Educational status, n (%)	Unable to read and write	13 (9.70)	5 (3.70)	0.028
	Able to read and write	21 (15.70)	10 (7.50)	
	Primary school	37 (27.60)	43 (32.10)	
	High school and above	63 (47.00)	76 (56.70)	
Residence, n (%)	Urban	113 (84.30)	107 (79.90)	0.141
	Rural	21 (15.70)	27 (20.10)	
BMI (kg/m^2^), median (IQR)		24.50 (22.10–27.70)	22.50 (21.1–24.10)	<0.001
WC (cm), median (IQR)		79.00 (74.00–85.00)	75.00 (70.0–79.00)	<0.001
WHR, mean ± SD		0.88±0.10	0.86±0.10	<0.001
Systolic BP (mmHg), median (IQR)		120 (110–130)	120 (110–120)	<0.001
Diastolic BP (mmHg), median (IQR)		80 (70–80)	70 (70–80)	0.002
FBG level (mg/dl), median (IQR)		159.20 (136.00–185.00)	_	
Duration of DM, median (IQR)		7.00 (4.00–9.00)	_	

**Note**: p-value <0.05 is considered as statistically significant.

### Comparison of hematological parameters of the study participants

There is statistically significant difference between T2DM patients and control group with respect to WBC (*P* = 0.004), absolute neutrophil (*P* = 0.02), absolute lymphocyte (*P*<0.001), absolute eosinophil (*P*<0.001), and absolute basophil counts (*P*<0.001). Regarding the RBC indices, the mean Hgb in patients with T2DM patients (15.70±1.20) was significantly lower than the control (16.2±1.30) group (P = 0.007). The median (IQR) of RDW was significantly increased in T2DM groups than the control group (P<0.001). Additionally, there were significantly higher mean platelet count (P = 0.013) and mean MPV (P = 0.010) in the T2DM group than the control group (Table 2).

**Table 2:** Comparison of hematological parameters of type 2 diabetic adult patients and control group, at Debre Berhan Referral Hospital, Northeast Ethiopia, 2020 (n = 268).

Parameters	T2DM (n = 134)	Control (n = 134)	p-value
WBC (10^3^/μl), mean ± SD	7.01±1.74	6.50±1.34	0.004
Neu (10^3^/μl), mean ± SD	4.14±1.51	3.80±0.96	0.020
Lymph (10^3^/μl), mean ± SD	2.07±0.62	1.86±0.54	<0.001
Mon (10^3^/μl), median (IQR)	0.60 (0.40–0.70)	0.50 (0.4–00.70)	0.150
Eos (10^3^/μl), median (IQR)	0.20 (0.10–0.30)	0.10 (0.10–0.20)	<0.001
Bas (10^3^/μl), median (IQR)	0.10 (0.00–0.01)	0.00 (0.00–0.00)	<0.001
RBC (10^6^/μl), mean ± SD	5.10±0.45	5.20±.0.50	0.100
Hgb (g/dl), mean ± SD	15.70±1.20	16.20±1.30	0.007
HCT (%), median (IQR)	46.40 (43.50–48.60)	46.60 (43.60–49.20)	0.206
MCV (fl), median (IQR)	90.10 (88.40–94.00)	90.80 (87.20–93.60)	0.917
MCH (Pg), median (IQR)	31.00 (29.80–32.20)	31.10 (30.20–32.30)	0.307
MCHC (%), mean ± SD	34.20±1.00	34.50±1.00	0.052
RDW (%), median (IQR)	14.00 (13.40–14.70)	13.50 (13.10–14.00)	<0.001
Platelet(10^3^/μl), mean ± SD	262.80±57.20	247.30±43.10	0.013
MPV (fl), mean ± SD	8.50±1.10	8.20±0.80	0.010

**Note**: p-value <0.05 is considered as statistically significant.

### Prevalence of hematological abnormality in type 2 diabetic adult patients

The prevalence of anemia in the current study among T2DM patients was 24 (17.90%). Leukocytosis was observed in 5 (3.70%) of T2DM patients. Additionally, out of 134 study subjects, 2 (1.50%) had neutrophilia and 5 (3.70%) had neutropenia. On the other hand, lymphocytosis and lymphopenia have occurred in 3 (2.20%) and 2 (1.50%) of T2DM patients, respectively. Moreover, 5 (3.70%) patients had monocytopenia, 6 (4.50%) had eosinophilia, 7 (5.20%) had basophilia, and 2 (1.50%) had thrombocytopenia.

### Correlations of hematological parameters with anthropometric and clinical variables among type 2 diabetic adult patients

In the correlation analysis, RBC count (rho = 0.18, p = 0.04) and RDW (rho = 0.26, p = 0.003) showed a statistically significant correlation with the duration of diabetes. Lymphocyte count showed significant and weak positive correlation with SBP (rho = 0.20, p = 0.02) and DBP (rho = 0.22, p = 0.01). Basophil count (rho = 0.18, p = 0.03) and MCH (rho = 0.19, p = 0.03) also showed a weak positive and significant correlation with DBP. Platelet count achieved a statistically significant negative correlation with BMI (rho = -0.19, p = 0.02) and WHR (rho = -0.20, p = 0.01). Additionally, MPV (rho = 0.19, *P* = 0.02) achieved a statistically significant correlation with BMI. In the current study, a statistically significant correlation was not observed between hematological parameters and fasting blood glucose levels.

### Factors associated with anemia among type 2 diabetic adult patients

Categorical variables were created from hemoglobin after the hemoglobin was adjusted for an average altitude of 3000 meters with a factor of 1.9 based on the World Health Organization (WHO) recommendation [30]. Associations of anemia with the predictor variables were evaluated using univariable and multivariable logistic regression analysis in diabetic individuals. In the bivariable logistic regression analysis gender, duration of DM, anti-DM drug, and milk consumption were associated with anemia with a p-value of less than 0.2. Variables with a p-value of less than 0.2 were included in the multivariable logistic regression model; duration of diabetes, and milk consumption were significantly associated with anemia. Patients who had been diagnosed with diabetes for 7 years or above were 3 times more likely to have anemia compared to those who have had it for less than 7 years (AOR = 3.05, 95% CI = 1.12–8.34). Type 2 diabetic patients with milk consumption were 4.6 times more likely to have anemia compared to those who do not consume milk (AOR = 4.60, 95%CI = 1.50–14.00) (Table 3).

**Table 3:** Factors associated with anemia among type 2 diabetic adult patients at Debre Berhan Referral Hospital, Northeast Ethiopia, 2020 (n = 134).

Variables	Categories	Anemia		COR (95% CI)	P-Value	AOR (95% CI)	P-Value
		Yes (%)	No (%)				
Age	18–41	11 (45.5)	49 (44.5)	1.05 (0.43–2.55)	0.90		
	42–65	13 (54.5)	61 (55.5)	1.00			
sex	Male	18 (75)	67 (60.9)	1.00		1.00	
	Female	6 (25)	43 (39.1)	0.5 (0.20–1.42)*	0.20	0.35 (0.12–1.03)	0.06
Residence	Urban	22 (91.7)	94 (85.5)	1.00			
	Rural	2 (8.3)	16 (15.5)	0.53 (0.11–2.50)	0.42		
BMI	Normal	12 (50.0)	60 (54.5)	1.00			
	Abnormal	12 (50.0)	50 (45.5)	1.2 (0.49–2.90)	0.69		
Glycemic control	Good control	8 (33.3)	48 (43.6)	1.00			
	Poor control	16 (66.7)	62 (56.4)	1.55 (0.61–3.92)	0.36		
Anti-DM drug	Metformin	19 (79.2)	99 (90)	1.00		1.00	
	Metformin+Sulfonylurea	5 (20.8)	11 (10)	2.40 (0.73–7.60)*	0.14	2.40 (0.58–7.04)	0.27
Duration of DM	< 7 years	7 (29.2)	57 (51.8)	1.00		1.00	
	≥ 7 years	17 (70.8)	53 (48.2)	2.61 (1.00–6.80)*	0.05	3.05 (1.12–8.30)*	0.03
Physical exercise	No	14 (58.3)	37 (33.6)	1.00			
	Yes	10 (41.7)	73 (66.4)	0.36(0.14–0.90)	0.25		
Vegetable consumption	No			1.09 (0.33–3.60)	0.88		
	Yes			1.00			
Egg consumption	No	11 (45.8)	51 (46.4)	1.00(0.40–2.37)	0.96		
	Yes	13 (54.2)	59 (53.6)	1.00			
Meat consumption	No	7 (29.2)	42 (38.2)	0.67(0.26–1.74)	0.41		
	Yes	17 (70.8)	68 (61.8)	1.00			
Milk consumption	No	5 (20.8)	52 (47.3)	1.00		1.00	
	Yes	19 (79.2)	58 (52.7)	3.4 (1.2–9.80)*	0.02	4.6 (1.50–14.00)*	0.008
Tea/coffee consumption	No	3 (12.5)	26 (23.6)	1.00			
	Yes	21 (87.5)	84 (76.4)	2.17 (0.60–7.84)	0.24		

**Note:** COR: Crud Odds Ratio, AOR: Adjusted Odds Ratio, CI: Confidence Interval

*: Statistically significant association.

## Discussion

It has been documented that hematological change is a common complication of DM and represents a significant and under-recognized burden in these patients [31]. In the present study, there was a statistically significant difference in total WBC count, absolute count of neutrophil, lymphocyte, eosinophil, basophil, platelet count, Hgb, RDW and MPV) of T2DM patients compared to control. The mean RBC count was lower in T2DM patients as compared to the control group, but the difference was not statistically significant. This finding is in coherence with the report of studies conducted in India [32], Libya [33], Sudan [21], and Addis Ababa, Ethiopia [34]. The possible explanation for decreased RBCs count might be that persistent hyperglycemia causes increased production of ROS and nonenzymatic glycosylation of Hgb and RBC membrane proteins leading to reduced deformability, increased aggregation, and accelerated aging of RBCs [10,35,36]. These changes in RBCs are also shown to markedly increase blood viscosity that adversely affects the microcirculation in diabetes, leading to microangiopathy [35]. In contrary to our findings, studies carried out in Pakistan [22] and Gondar, northwest Ethiopia [23] reported higher RBC count and Hgb concentration in T2DM patients than controls. This might be explained by the effect of insulin resistance, which is associated with the stimulation of erythroid progenitors increasing RBC count, and increased levels of Hgb and HCT [37].

Regarding RDW, the present study revealed that RDW values were significantly higher in T2DM patients than control groups. This finding is in harmony with the findings of previous studies in Pakistan [22], Saudi Arabia [24], Addis Ababa, Ethiopia [34], and Gondar, northwest Ethiopia [23]. Higher RDW indicates the presence of heterogeneity among the circulating RBCs, which is related to the impairment of erythropoiesis and degradation of RBCs [38]. Chronic inflammation and increased level of oxidative stress are common in diabetes and they are known to reduce RBCs’ survival that results in variation in RBCs size and decreased RBCs count [39].

In the current study, we observed that the Hgb as an index of anemia was significantly lower among T2DM patients as compared to controls. This finding is supported by previous studies carried out in Bangladesh [40], India [32], Libya [33], and Nigeria [41] that have been reported significantly lower Hgb in T2DM patients than the control group. The prevalence of anemia was 17.9% (95% CI: 11.5–24.5), which is comparable with studies conducted in Saudi Arabia 22% [6], Australia 17.8% [7], and Sudan 18.3% [21]. In contrast, our prevalence estimate was lower than those reported by studies conducted in India 71.4% [32], Nigeria 45.2% [41], and Dessie, northeast Ethiopia 26% [42]. This discrepancy might be due to a difference in the characteristics of the study population and sample size variation. In our study the participants were adults and most of them were males.

According to the WHO classification of anemia public health significance in populations [30], the current study showed a mild (5.0–19.9%) prevalence of anemia and a public health problem among T2DM. The etiology of anemia in T2DM is multifactorial and includes chronic hyperglycemia, inflammation, oxidative stress, AGEs, nutritional deficiencies, drugs, and hormonal changes in addition to kidney disease [36,43]. Chronic inflammation in diabetes is characterized by increased inflammatory cytokines such as interleukin 6 (IL-6), and interleukin (IL-1). These pro-inflammatory cytokines are thought to changes the sensitivity of erythroid progenitors to erythropoietin and promote apoptosis of immature RBCs and decrease the number of circulating RBCs resulting anemia of inflammation [43]. Whatever the cause, the consequences of anemia complicating diabetes appear adverse, including evidence of increased all-cause and cardiovascular mortality [7,43].

On logistic regression analysis, the duration of DM was significantly associated with anemia similar to the studies from Nigeria [41]. Patients who had DM for more than 7 years were 3 times more likely to have anemia compared to those who had DM for 7 years or less. Evidence suggested that the longer the duration of the disease the higher the inflammatory process, resulting in increased IL-6 with anti-erythropoietic effect, causing a decrease in the number of circulating RBCs and consequently causing a reduction of circulating hemoglobin [43]. Milk consumption (AOR = 4.60, 95% CI = 1.50–14.0) was significantly associated with anemia. Milk consumption might bring this effect by altering the absorption of iron due to its high calcium and casein contents as well as the low content of iron and folate in milk.

Regarding WBC indices, the present study demonstrated that total WBC count, absolute neutrophil count, and absolute lymphocyte count were significantly higher in the T2DM group compared to the control group. This is in corroboration with the report of previous studies conducted in Turkey [20], Bangladesh [40], Libya [33], and Gondar, northwest Ethiopia [23]. Absolute eosinophil and basophil count also showed a significant increase in the diabetic group than the control group in consonance with the study conducted in Saudi Arabia [24] and Bangladesh [40]. The pattern of WBC disturbances in T2DM patients are not widely available in the literature; in the current study 3.7% had leukocytosis, 1.5% had neutrophilia, 4.5%) had eosinophilia, and 5.2% had basophilia.

The mechanism underlying this increase of the total and differential WBC counts in T2DM patients might be explained by the effect of hyperglycemia and the pathogenesis of T2DM. The available biological data have strongly suggested that T2DM is an inflammatory disease [44]. Increased WBC count is a classical marker of inflammation and evidence from epidemiological studies suggests an association between WBC count and diabetes risk [15]. Although defects in insulin action on the peripheral tissues lead to a chronic low-grade inflammatory state and induce the secretion of proinflammatory cytokines, which promote differentiation and maturation of leukocytes [15,45]. Additionally, in hyperglycemic state leukocytes are activated by AGEs, oxidative stress, and cytokines that increase the state of inflammations and the development of vascular complications in diabetes [12,36]. Neutrophils and monocytes are also suggested to be a marker of inflammation, which is associated with the progression of complications [20,36].

In the present study, the differential white cell counts showed that majority of 127 (94.8%) of T2DM patients had a normal neutrophil count and 5 (3.7%) had neutropenia. Diabetic neutrophils have been associated with impaired deformability, chemotaxis, phagocytosis, bactericidal activity, and they also die sooner than normal [46], which might also explain the neutropenia in the present study. Regarding absolute monocyte count, the present study showed that there was no significant difference between the diabetic and control group. This is in agreement with the study conducted in Ethiopia [23,34] and contrary to a study conducted in Turkey [20] and Bangladesh [40]. Additionally, 5 (3.7%) of T2DM patients had monocytopenia. The reason for this might be that several stimuli, including pro-inflammatory as well as metabolic stimuli, increase the recruitment of monocytes to peripheral tissues, where they differentiate to macrophages and dendritic cells. The destination of monocytes is therefore not the bloodstream and hence peripheral enumeration is not representative of monocyte tissue presence [44].

In this study, analysis of the platelet indices demonstrated that MPV and platelet counts were significantly higher in T2DM compared to the controls. Similar to this study, studies conducted in Nigeria [47] and Ethiopia [34] found that platelet counts were significantly higher in the diabetes group as compared to the controls. The increased MPV among T2DM patients in this study is also in agreement with several studies [22–24,34]. The reason might be that platelet counts and MPV are indicators of thrombotic potential and risk of vascular complications in diabetes. There might be a release of S100A8/A9 by neutrophils which triggers IL-6 production and thrombopoietin synthesis from hepatocytes, leading to bone marrow stimulation to recruit greater numbers of reticulated platelets, which are associated with both atheroprogression and atherothrombosis [48].

The other reason might be due to differences in platelet function between diabetic and control individuals. Evidence suggests that platelets from patients with T2DM have increased reactivity and baseline activation compared to healthy controls [49]. The function of platelet and its size are said to be related and large circulating platelets are reflected by higher MPV which is the marker of the average size, and platelet activity [50]. Diabetic platelets are larger with denser granules and they are enzymatically and functionally hyperactive to produce more prothrombotic factors like thromboxane A2, platelet factor 4, serotonin, and P-selectin than smaller platelets and hence cause an increased tendency to thrombotic events [51]. Platelet hyperactivity in diabetics patients is also attributed to a multitude of factors including insulin resistance, oxidative stress, endothelial dysfunction, inflammation, and hyperglycemia [11,49,52].

Additionally, the present study assessed the correlation of hematological parameters with several cardio-metabolic risk factors. In our study, RBC and RDW were correlated with the duration of diabetes. This relationship might be explained by the effect of chronic hyperglycemia in which lower RBC count and elevated RDW are frequently observed in diabetics with a long duration of disease and vascular complication [53]. Lymphocyte count showed significant and weak positive correlation with SBP and, similar to the study conducted in Turkey [20] and Gondar, Ethiopia [23]. Lymphocyte, and basophil count, and MCH significantly correlated with DBP.

The association between blood pressure and hematological parameters might due to the development of endothelial dysfunction and hypertension in diabetes mellitus. Evidence suggested that hyperglycemia triggers damage to the vascular bed by several cellular mechanisms including accumulation of ROS and AGEs; creating an imbalance between vasodilators and vasoconstrictors [11]. This condition can lead to increased vasoconstriction and vascular remodeling ultimately affecting the blood cells. In respect to the correlation of hematological parameters with body adiposity, platelet count was statistically correlated with BMI and WHR. Moreover, MPV achieved a statistically significant correlation with BMI. A similar study on adult Nigerian people with T2DM found that platelet count and MPV were statistically correlated with BMI. The observed relationship might be due to the fact that obesity is associated with systemic inflammation that could play a role in platelet activation and the production of larger platelets [54].

According to our extensive literature, there has been no comprehensive study assessing the hematological parameters in type 2 diabetes patients; which is addressed by the current study. Despite its strength, our study has some limitations. One limitation of this study is that we cannot determine a cause-effect relationship due to the cross-sectional nature of our study design. Also, morphological and coagulation profile study were not assessed. Another limitation is that it was a single Hospital-based study, thus the observed prevalence of anemia may not reflect its actual burden among patients with type 2 diabetes in the general community.

### Conclusion and recommendations

In the present study, there was a statistically significant variation in the hematological parameters (total WBC count, absolute count of neutrophil, lymphocyte, eosinophil, and basophil, platelet count, Hgb, RDW and MPV) of T2DM patients compared to control. This study also highlights that anemia was a common hematological change among T2DM patients and it was a mild public health problem in our clinical practice. Of the examined patients, nearly one out of five diabetic patients were anemic. Longer duration of diabetes and milk consumption increased the likelihood of anemia. Platelet count and MPV were significantly correlated with anthropometric measurements. Red blood cells count and RDW were correlated with the duration of diabetes. Thus, assessment of the hematological changes in T2DM patients will be of paramount importance enabling the clinician to establish an effective and early therapeutic intervention to prevent the occurrence of major complications. Regular screening of hematological parameters should be considered for proper management of type 2 diabetic patients. Close attention should also be given to the duration of diabetes and dietary practice with respect to hematological abnormality. A longitudinal study with a larger sample size would be superior to assess the problem very well. Moreover, morphological and coagulation profile study would be worth considering for T2DM patients.

## Supporting information

S1 File Questionnaire. (DOCX)

S2 File Hematological parameters of T2DM &control (merged) raw data. (XLSX)

S3 File Hematological parameters of T2DM raw data. (XLSX)

## Abbreviations

AGE: Advanced Glycation End Products
AOR: Adjusted Odds Ratio
BMI: Body Mass Index
BP: Blood Pressure
CBC: Complete Blood Count
COR: Crude Odds Ratio
CVD: Cardiovascular Disease
DBP: Diastolic Blood Pressure
DM: Diabetes Mellitus
EDTA: Ethylene Di-amino Tetra Acetic acid
FBG: Fasting Blood Glucose
HbA1c: Glycated hemoglobin
HC: Hip Circumference
Hct: Hematocrit
Hgb: Hemoglobin
IL-1: Interleukin 1
IL-6: Interleukin 6
IQR: Inter Quartile Range
MCH: Mean Corpuscular Hemoglobin
MCHC: Mean Cell Hemoglobin Concentration
MCV: Mean Corpuscular Volume
MPV: Mean Platelet Volume
PLT: Platelet
RBC: Red Blood Cell
RDW: Red blood cell Distribution Width
ROS: Reactive Oxygen Species
SBP: Systolic Blood Pressure
SD: Standard Deviation
T2DM: Type 2 Diabetes Mellitus
WBC: White Blood Cell
WC: Waist Circumference
WHO: World Health Organization
WHR: Waist to Hip Ratio
