# Supplementary material for: Hematological parameters of type 2 diabetic adult patients at Debre Berhan Referral Hospital, Northeast Ethiopia: A comparative cross-sectional study
Source: PLoS One. 2021 Jun 14;16(6):e0253286. doi: 10.1371/journal.pone.0253286 (PMC8202906; doi:10.1371/journal.pone.0253286)
Supplement: S1 File — (DOCX) [file pone.0253286.s001.docx]

## Questionnaire

**Questionnaire for T2DM group**

Data collection questionnaire designed to conduct a study on hematological parameters of type 2 diabetic adult patients at Debre Berhan Referral Hospital, Northeast Ethiopia, 2020.

**Instructions**: This questionnaire contains a question, which is pertinent to the research

objectives. You are kindly requested to answer all the questions as much as possible.

Does the patient fulfill all inclusion criteria and no exclusion criteria? 1. No 2. Yes

**Identification code**_______________

| **S. No** | **Questions** | **Possible answers** |
| --- | --- | --- |
|  | **Part I.** Sociodemographic variables | |
|  | Age (in years) | __________ |
|  | Gender | 1. Male 2. Female |
|  | Educational level | 1. Unable to read & write |
|  |  | 1. Can read and write |
|  |  | 1. Elementary school |
|  |  | 1. High school & above |
|  | Residency | 1. Urban 2. Rural |
|  | Occupation | 1. Government employee 2. Private employee 3. Private worker 4. Other |
|  | **Part II**. Anthropometric and BP measurements | |
|  | Weight | _____kg |
|  | Height | ______m |
|  | BMI | ______ kg / (m)^2^ |
|  | Waist Circumference (WC) | ______cm |
|  | Hip Circumference (HC) | ______cm |
|  | WHR | _______ |
|  | Blood pressure (BP) | Systolic_____mmHg  Diastolic_____ mmHg |

|  | **Part** **III**: Behavioral variables | | |
| --- | --- | --- | --- |
| 301. | Do you perform physical exercise | 1. No 2. Yes | |
| 302. | Do you consume green leafy vegetables? | 1. No 2. Yes | If 1, skip to Q304 |
| 303. | If yes for Q302. How many times per week? | 1. One time 2. Two-three times 3. Above 3 times | |
| 304. | Do you consume eggs? | 1. No 2. Yes | If 1, skip to Q306 |
| 305. | If yes for Q304. How many times per week? | 1. One time 2. Two-three times 3. Above 3 times | |
| 306. | Do you consume red meat | 1. No 2. Yes | If 1, skip to Q308 |
| 307. | If yes for Q306. How many times per week? | 1. One time 2. Two-three times 3. Above 3 times | |
| 308. | Do you consume milk products? | 1. No 2. Yes | If 1, skip to 310 |
| 309. | If yes for Q308. How many times per week? | 1. One time 2. Two-three times 3. Above 3 times | |
| 310. | Do you have a habit of drinking coffee or tea after a meal? | 1. No 2. Yes | |
| Data collector name _________________ Date ___________________Signature____________ | | | |

**Questionnaire for the control group (blood donors)**

Does the participant fulfill all donors’ criteria? 1. No 2. Yes

**Identification code**_______________

| **S. No** | **Questions** | **Possible answers** |
| --- | --- | --- |
|  | **Part I.** Sociodemographic variables | |
| 101. | Age (in years) | __________ |
| 102. | Gender | 1. Male 2. Female |
| 103. | Educational level | 1. Unable to read & write |
|  |  | 1. Can read and write |
|  |  | 1. Elementary school |
|  |  | 1. High school & above |
| 104. | Residency | 1. Urban 2. Rural |
| 105. | Occupation | 1. Government employee 2. Private employee 3. Private worker 4. Other |
|  | **Part II**. Anthropometric and BP measurements | |
| 201. | Weight | _____kg |
| 202. | Height | ______m |
| 203. | BMI | ______ kg / (m)^2^ |
| 204. | Waist Circumference (WC) | ______cm |
| 205. | Hip Circumference (HC) | ______cm |
| 206. | WHR | _______ |
| 207. | Blood pressure (BP) | Systolic_____mmHg  Diastolic_____ mmHg |
| Data collector name _________________ Date ___________________Signature____________ | | |

## Checklist

Checklist prepared to collect clinical variables from the medical record of type 2 diabetic patients at Debre Berhan Referral Hospital northeast Ethiopia.

**Identification code**_______________

| S.no | Clinical variables | Possible options |
| --- | --- | --- |
| 101. | Oral hypoglycemic therapy used | 1. Metformin 2. Sulfonylureas 3. Metformin+ Sulfonylureas |
| 102. | Duration of oral hypoglycemic therapy used | _____________ |
| 103. | Duration of DM since diagnosis (in years) | _______ |
| 104. | Fasting blood glucose level in the last 2 months | 1. Month1_____mg/dl 2. Month2____mg/dl |
| Data collector name _______________ Date ___________________Signature____________ | | |
